# Supplementary material for: What Can One Minute of the Day Tell about Physical Activity?
Source: Int J Environ Res Public Health. 2023 Sep 28;20(19):6852. doi: 10.3390/ijerph20196852 (PMC10572772; doi:10.3390/ijerph20196852)
Supplement: Supplementary file 1 [file ijerph-20-06852-s001.zip › ijerph-2586547-supplementary.pdf]

Table S1. Mean (SD, range) values are presented for body mass index (BMI) and age across cardiorespiratory fitness (CRF) deciles for both men and women. The CRF groups are labelled from 0 for the lowest CRF group to 9 for the highest CRF group and the group specific range of the maximum oxygen uptake (VO_2max) is shown in the brackets.

CRF group (VO_2max range in ml/kg/min)	BMI (kg/m^2)	Age (years)
Men		
0 (11.2–28.4)	31.8 (5.5, 21.0–48.7)$^{1–9}$	58.4 (11.5, 22–70)$^{2–9}$
1 (28.4–31.2)	28.6 (3.5, 21.3–41.4)$^{0,3–9}$	54.9 (1.02, 27–70)$^{3–9}$
2 (31.2–33.7)	27.7 (3.3, 20.6–38.9)$^{0,4–9}$	52.7 (12.6, 20–70)$^{0,4–9}$
3 (33.7–35.7)	27.3 (3.3, 19.0–35.8)$^{0,1,7–9}$	49.0 (12.4, 24–69)$^{0,1,5–9}$
4 (35.7–37.5)	26.3 (3.1, 14.9–33.7)$^{0–2,8,9}$	47.9 (11.9, 26–69)$^{0–2,5–9}$
5 (37.5–39.0)	26.0 (3.2, 18.8–33.7)$^{0–2,8,9}$	41.8 (13.1, 20–69)$^{0–3,7–9}$
6 (39.0–40.9)	25.7 (2.7, 18.0–32.9)$^{0–2,8,9}$	41.4 (10.8, 21–69)$^{0–4,9}$
7 (40.9–42.6)	25.3 (2.7, 18.9–33.3)$^{0–3,9}$	36.2 (11.1, 20–69)$^{0–5,9}$
8 (42.6–45.3)	24.6 (2.7, 16.7–32.0)$^{0–6}$	36.8 (10.0, 21–65)$^{0–5,9}$
9 (45.3–53.4)	23.1 (2.4, 18.1–30.7)$^{0–7}$	29.8 (8.1, 20–56)$^{0–8}$
Women		
0 (9.9–24.6)	33.7 (4.3, 22.3–47.3)$^{1–9}$	54.7 (11.8, 27–70)$^{3–9}$
1 (24.6–27.9)	29.5 (3.5, 20.8–43.9)$^{0,2–9}$	53.6 (11.5, 24–70)$^{4–9}$
2 (27.9–30.3)	27.5 (3.0, 19.2–35.3)$^{0,1,3–9}$	51.4 (12.8, 20–69)$^{4–9}$
3 (30.3–32.3)	26.1 (2.7, 19.6–39.6)$^{0–2,5–9}$	48.9 (13.4, 20–69)$^{0,5–9}$
4 (32.3–33.8)	25.1 (2.5, 18.3–32.9)$^{0–2,6–9}$	45.8 (13.4, 20–69)$^{0–2,7–9}$
5 (33.8–35.1)	24.1 (2.5, 17.3–31.5)$^{0–3,7–9}$	44.5 (13.5, 20–69)$^{0–3,7–9}$
6 (35.1–36.7)	23.4 (2.2, 18.4–32.3)$^{0–4,8,9}$	41.6 (13.8, 20–69)$^{0–3,8,9}$
7 (36.7–38.4)	22.8 (2.1, 17.4–30.0)$^{0–5,9}$	38.5 (11.9, 20–69)$^{0–5,9}$
8 (38.4–40.3)	22.2 (2.0, 17.7–32.5)$^{0–6,9}$	35.6 (11.3, 20–68)$^{0–6}$
9 (40.4–48.6)	20.7 (1.8, 16.5–26.9)$^{0–8}$	33.2 (9.9, 20–69)$^{0–7}$

The superscripted number indicates a statistically significant difference ($p<0.05$) from the specified CRF decile group. An independent-samples Kruskal-Wallis's test with a Bonferroni post-hoc test was used to compare the differences between the CRF groups.

Table S2. Mean (SD) values are presented for the number of hours containing activity and peak intensities across cardiorespiratory fitness (CRF) deciles for both men and women. The CRF deciles are labelled from 0 for the lowest CRF group to 9 for the highest CRF group.

CRF decile	Daily peak intensity in metabolic equivalents (MET), oxygen uptake reserve (VO_2R) and the mean individual coefficient of variation (CV)			The number of hours containing at least one physical activity bout which intensity was over the threshold below in metabolic equivalents (MET)			The number of hours containing at least one physical activity bout which intensity was over the threshold below in oxygen uptake reserve (VO_2R)		
	peak MET	peak VO_2R	CV	3 MET	6 MET	8.8 MET	40 % VO_2R	60 % VO_2R	90 % VO_2R
Men									
0	4.7 (0.8)$^{1–9}$	65 % (18 %)$^{1–9}$	13 % (10 %)$^{3–9}$	7.3 (2.8)$^{1–9}$	0.10 (0.31)$^{2–9}$	0.02 (0.09)$^{2–9}$	5.4 (2.9)$^{1–9}$	1.46 (1.85)$^{1–9}$	0.22 (0.75)
1	5.1 (0.7)$^{0,3–9}$	54 % (9 %)$^{0,4–8,9}$	13 % (10 %)$^{3–9}$	8.4 (2.6)$^{0,5–9}$	0.17 (0.38)$^{3–9}$	0.02 (0.12)$^{3–9}$	3.3 (1.8)$^{0,3–9}$	0.34 (0.51)^{0}	0.04 (0.18)$^{6–8}$
2	5.3 (0.8)$^{0,5–9}$	51 % (10 %)^{0,9}	17 % (14 %)$^{6–9}$	8.8 (2.6)$^{0,6–9}$	0.22 (0.33)$^{0,3–9}$	0.04 (0.09)$^{0,3–9}$	2.7 (1.7)$^{0,4–9}$	0.24 (0.32)^{0}	0.04 (0.10)$^{6–8}$
3	5.7 (1.1)$^{0,1,6–9}$	52 % (13 %)^{0}	18 % (12 %)^{0,1,7,9}	9.0 (2.4)$^{0,6–8}$	0.40 (0.51)$^{0–2,6–9}$	0.10 (0.25)$^{0–2,6–9}$	2.3 (1.3)$^{0,1,6–9}$	0.27 (0.40)^{0}	0.10 (0.23)^{7}
4	5.6 (1.1)$^{0,1,6–9}$	49 % (12 %)^{0,1}	17 % (12 %)$^{0,1,6–9}$	8.8 (2.6)$^{0,6–9}$	0.36 (0.53)$^{0,1,6–9}$	0.09 (0.22)$^{0,1,6–9}$	1.8 (1.3)$^{0–2,9}$	0.20 (0.33)$^{0,6–8}$	0.07 (0.18)
5	5.9 (1.2)$^{0–2,7–9}$	50 % (12 %)^{0,1}	19 % (13 %)^{0,1,9}	9.2 (2.3)^{0,1}	0.49 (0.61)$^{0–2,7–9}$	0.16 (0.28)$^{0–2,7–9}$	1.8 (1.3)$^{0–2,9}$	0.25 (0.35)^{0}	0.10 (0.23)
6	6.2 (1.3)$^{0–4,9}$	50 % (12 %)^{0,1}	24 % (15 %)$^{0–2,4}$	10.0 (2.5)$^{0–4}$	0.61 (0.6)$^{0–4,9}$	0.20 (0.31)$^{0–4,9}$	1.6 (1.2)$^{0–3,9}$	0.33 (0.41)^{0,4}	0.11 (0.23)^{1,2}
7	6.7 (1.6)$^{0–5}$	52 % (15 %)^{0}	24 % (14 %)$^{0–4}$	10.0 (2.3)$^{0–4}$	0.83 (0.81)$^{0–5}$	0.32 (0.44)$^{0–5}$	1.5 (1.2)$^{0–3}$	0.39 (0.46)^{0,4}	0.13 (0.27)$^{1–3}$
8	6.8 (1.8)$^{0–5}$	51 % (16 %)^{0,1}	22 % (12 %)$^{0–2,4}$	10.0 (2.4)$^{0–4}$	0.89 (0.89)$^{0–5}$	0.32 (0.49)$^{0–5}$	1.3 (1.1)$^{0–3}$	0.40 (0.55)^{0,4}	0.15 (0.36)^{1,2}
9	7.0 (1.8)$^{0–6}$	48 % (14 %)$^{0–2}$	24 % (13 %)$^{0–5}$	9.8 (2.4)$^{0–2,4}$	1.08 (1.00)$^{0–6}$	0.37 (0.61)$^{0–6}$	1.0 (0.9)$^{0–6}$	0.38 (0.63)^{0}	0.11 (0.26)
Women									
0	4.8 (0.8)$^{2–9}$	76 % (20 %)$^{1–9}$	13 % (10 %)$^{3–9}$	6.8 (2.6)$^{1–9}$	0.13 (0.34)$^{2–9}$	0.02 (0.08)$^{3–9}$	6.8 (3.0)$^{1–9}$	2.66 (2.09)$^{1–9}$	0.41 (0.95)$^{1–6}$
1	5.0 (0.7)$^{3–9}$	62 % (12 %)$^{0,2–9}$	14 % (10 %)$^{5–9}$	7.6 (2.6)$^{0,4–9}$	0.18 (0.37)$^{3–9}$	0.02 (0.06)$^{4–9}$	4.7 (2.2)$^{0,2–9}$	1.13 (1.14)$^{0,2–9}$	0.07 (0.14)^{0,8,9}
2	5.3 (0.9)$^{0,3–9}$	59 % (13 %)^{0,1,6,9}	15 % (10 %)$^{5–9}$	7.7 (2.6)$^{0,4–9}$	0.26 (0.45)$^{0,3–9}$	0.04 (0.11)$^{5–9}$	3.5 (1.8)$^{0,1,6–9}$	0.74 (0.89)$^{0,1,4–7}$	0.08 (0.18)^{0,8,9}
3	5.6 (1.0)$^{0–2,5–9}$	58 % (13 %)^{0,1}	17 % (11 %)$^{0,7–9}$	8.2 (2.4)$^{0,6–9}$	0.42 (0.61)$^{0–2,5–9}$	0.08 (0.23)$^{0,7–9}$	3.3 (1.6)$^{0,1,6–9}$	0.57 (0.71)^{0,1}	0.11 (0.24)^{0}
4	5.8 (1.1)$^{0–2,7–9}$	57 % (13 %)^{0,1}	17 % (10 %)$^{0,7–9}$	8.7 (2.4)$^{0–2}$	0.47 (0.59)$^{0–2,7–9}$	0.08 (0.20)$^{0,1,7–9}$	3.1 (1.6)$^{0,1,7–9}$	0.43 (0.57)$^{0–2}$	0.09 (0.22)^{0}
5	6.0 (1.0)$^{0–3,7–9}$	56 % (12 %)^{0,1}	18 % (11 %)$^{0–2,8,9}$	8.8 (2.5)$^{0–2}$	0.62 (0.67)$^{0–3,8,9}$	0.10 (0.20)$^{0–2,7–9}$	3.0 (1.7)^{0,1,8,9}	0.42 (0.52)$^{0–2}$	0.09 (0.18)^{0}
6	6.0 (1.1)$^{0–3,8,9}$	54 % (12 %)$^{0–2}$	19 % (11 %)$^{0–2,8,9}$	9.1 (2.4)$^{0–3}$	0.66 (0.70)$^{0–3,8,9}$	0.12 (0.25)$^{0–2,7–9}$	2.6 (1.6)$^{0–3,9}$	0.35 (0.45)$^{0–2}$	0.10 (0.23)^{0}
7	6.5 (1.3)$^{0–5,9}$	56 % (14 %)^{0,1}	22 % (11 %)$^{0–4}$	9.2 (2.4)$^{0–3}$	0.82 (0.83)$^{0–4,9}$	0.20 (0.35)$^{0–6,9}$	2.5 (1.3)$^{0–4}$	0.37 (0.46)$^{0–2}$	0.14 (0.30)
8	6.6 (1.5)$^{0–6}$	55 % (15 %)^{0,1}	21 % (10 %)$^{0–6}$	9.3 (2.1)$^{0–3}$	0.90 (0.79)$^{0–6}$	0.24 (0.36)$^{0–6}$	2.3 (1.3)$^{0–5}$	0.40 (0.48)^{0,1}	0.14 (0.27)^{1,2}
9	7.0 (1.5)$^{0–7}$	54 % (12 %)$^{0–2}$	24 % (11 %)$^{0–6}$	9.4 (2.3)$^{0–3}$	1.17 (1.05)$^{0–7}$	0.31 (0.41)$^{0–7}$	2.0 (1.4)$^{0–6}$	0.44 (0.47)^{0,1}	0.13 (0.26)^{1,2}

The superscripted number indicates a statistically significant difference ($p<0.05$) from the specified CRF decile group. An independent-samples Kruskal-Wallis's test with a Bonferroni post-hoc test was used to compare the differences between the CRF groups.
